# Molecular Pathways Involved in the Amelioration of Myocardial Injury in Diabetic Rats by Kaempferol

**DOI:** 10.3390/ijms18051001

**Published:** 2017-05-15

**Authors:** Kapil Suchal, Salma Malik, Sana Irfan Khan, Rajiv Kumar Malhotra, Sameer N. Goyal, Jagriti Bhatia, Shreesh Ojha, Dharamvir Singh Arya

**Affiliations:** 1 Cardiovascular Research Laboratory, Department of Pharmacology, All India Institute of Medical Sciences, New Delhi 110029, India; kapilsuchal@gmail.com (K.S.); malik.salma87@gmail.com (S.M.); drsanashaham@gmail.com (S.I.K.); rmalhotra.pharma2008@gmail.com (R.K.M.); jagriti2012@gmail.com (J.B.); 2 Department of Pharmacology, R.C. Patel Institute of Pharmaceutical Education and Research, Shirpur, Maharashtra 425405, India; goyal.aiims@gmail.com; 3 Department of Pharmacology and Therapeutics, College of Medicine and Health Sciences, United Arab Emirates University, Al Ain, Abu Dhabi 17666, UAE

**Keywords:** AGE-RAGE, apoptosis, inflammation, kaempferol, ischemia-reperfusion, diabetes

## Abstract

There is growing evidence that chronic hyperglycemia leads to the formation of advanced glycation end products (AGEs) which exerts its effect via interaction with the receptor for advanced glycation end products (RAGE). AGE-RAGE activation results in oxidative stress and inflammation. It is well known that this mechanism is involved in the pathogenesis of cardiovascular disease in diabetes. Kaempferol, a dietary flavonoid, is known to possess antioxidant, anti-apoptotic, and anti-inflammatory activities. However, little is known about the effect of kaempferol on myocardial ischemia-reperfusion (IR) injury in diabetic rats. Diabetes was induced in male albino Wistar rats using streptozotocin (70 mg/kg; i.p.), and rats with glucose level >250 mg/dL were considered as diabetic. Diabetic rats were treated with vehicle (2 mL/kg; i.p.) and kaempferol (20 mg/kg; i.p.) daily for a period of 28 days and on the 28th day, ischemia was produced by one-stage ligation of the left anterior descending coronary artery for 45 min followed by reperfusion for 60 min. After completion of surgery, rats were sacrificed and the heart tissue was processed for biochemical, morphological, and molecular studies. Kaempferol pretreatment significantly reduced hyperglycemia, maintained hemodynamic function, suppressed AGE-RAGE axis activation, normalized oxidative stress, and preserved morphological alterations. In addition, there was decreased level of inflammatory markers (tumor necrosis factor-α (TNF-α), interleukin-6 (IL-6), and NF-κB), inhibition of active c-Jun N-terminal kinase (JNK) and p38 proteins, and activation of Extracellular signal regulated kinase 1/2 (ERK1/2) a prosurvival kinase. Furthermore, it also attenuated apoptosis by reducing the expression of pro-apoptotic proteins (Bax and Caspase-3), Terminal deoxynucleotidyl transferase dUTP nick end labeling (TUNEL) positive cells, and increasing the level of anti-apoptotic protein (Bcl-2). In conclusion, kaempferol attenuated myocardial ischemia-reperfusion injury in diabetic rats by reducing AGE-RAGE/ mitogen activated protein kinase (MAPK) induced oxidative stress and inflammation.

## 1. Introduction

The morbidity and mortality due to myocardial infarction remains high especially in diabetic patients, despite the availability of numerous therapeutic modalities [1]. Clinical studies have also demonstrated that diabetes mellitus (DM) increases the susceptibility of myocardium to ischemia-reperfusion (IR) injury [2]. The mechanism of diabetes induced cardiovascular disease is still unknown. Previous studies have shown that uncontrolled hyperglycemia leads to synthesis of advanced glycation end products (AGEs) [3]. AGEs constitute a group of heterogeneous compounds that cumulate in diabetes due to a rise in reactive carbohydrate substrate availability, oxidative condition, as well as impaired detoxification [4]. There is a growing body of evidence that AGEs have a role in the pathogenesis of diabetic complications in various organs including the heart [5]. The mechanism by which AGE produces its effect in the pathogenesis of cardiovascular disease has been postulated to be direct as well as indirect. The direct effect of AGE is through an alteration of the cellular physiochemical property by the formation of cross linked macromolecules [6]. The indirect effect of AGE involves its interaction with the receptor for advanced glycation end products (RAGE). In normal healthy subjects, the expression of RAGE is low, however, in diseased states such as diabetes, inflammation, and cardiovascular pathology, there is an increased expression of RAGE [7]. Bucciarelli along with his colleagues demonstrated that cardiac IR led to the up regulation of RAGE [8]. Various studies have shown that a deficiency of RAGE inhibits the development of atherosclerosis in the diabetic myocardium [9]. Tikellis et al. (2008) investigated the relationship between RAGE and oxidative stress in vivo and showed that RAGE-deficient mice displayed reduced markers of oxidative stress [10].

Oxidative stress also leads to phosphorylation of the major signal transduction cascade molecule mitogen activated protein kinase (MAPK). The latter subsequently activates nuclear transcription factors including NF-κB which regulates gene expression of various cytokines and chemokines [11]. In a recent study, inhibition of the p38/c-Jun N-terminal kinase (JNK) MAPK pathway has been shown to improve diabetic cardiomyopathy by attenuating AGE-RAGE mediated oxidative stress and inflammation [12]. Since oxidative stress plays a critical role in the pathogenesis of IR injury, the suppression of RAGE or possibility of reducing glycation and AGEs can serve as an attainable target of halting the onset of diabetic complications. Numerous compounds both natural and pharmacological are under investigation for their possible therapeutic potential.

Flavonoids are polyphenolic compounds which exist as a component of the plant kingdom and serves as an important part of the human diet. The antioxidant and anti-inflammatory roles of these compounds have been studied extensively [13]. Kaempferol, a member of the flavonols, is abundantly found in tea, broccoli, apples, strawberries, and beans [14]. Kaempferol is also present in different medicinal plants like *Acacia nilotica* (L.) Delile, *Aloe vera* (L.) Burm.f., and *Crocus sativus* L. [15]. It has been reported to possess various pharmacological effects such as antioxidant [16], anti-inflammatory [17], anti-apoptotic [18], and anti-diabetic [19] in different experimental models. Kaempferol has been shown to exert an anti-inflammatory effect through inhibition of the MAPK pathway [17]. However, the effect of kaempferol on AGE-RAGE axis in diabetic rats still needs to be evaluated. Hence, the present study was undertaken to investigate whether kaempferol attenuates myocardial IR injury in diabetic rats through inhibition of the MAPK pathway and further to elucidate the role of AGE-RAGE inhibition in the cardioprotective effect of kaempferol in this condition.

## 2. Results

### 2.1. Mortality Rate

Total 14.7% mortality was observed due to diabetes, bleeding, or improper ligation of the left anterior descending (LAD) coronary artery. One rat died from the diabetic-control, three rats from diabetes + IR, and one rat from the treatment group. So, to equalize the number of rats in the experiment, further study was carried out on nine rats per group. From the nine rats in a given group, the hearts of six rats were used for estimation of the biochemical parameters (malondialdehyde (MDA), reduced glutathione (GSH), superoxide dismutase (SOD), and catalase (CAT)) and from the remaining three rats, a half part of the heart removed was used for Western blot analysis and the other half was used for histopathology, ultrastructural, and Terminal deoxynucleotidyl transferase dUTP nick end labeling (TUNEL) analysis.

### 2.2. Body Weight Measurement

The body weight in all experimental groups was assessed at the 1st, 7th, 14th, 21st, and 28th day. There was significant change in body weight in all groups as compared to their baseline values (*p* < 0.05). However, no significant change was observed among the experimental groups (Table 1).

### 2.3. Blood Glucose Assessment

Similar to body weight, the blood glucose level was assessed at the 1st, 7th, 14th, 21st, and 28th day. There was no significant change in fasting blood glucose level in the diabetic control and diabetes + IR groups in comparison to their baseline values. Pre-treatment with kaempferol (20 mg/kg) for 28 days decreased the blood glucose level as compared to the diabetic control and diabetes + IR group (Table 2).

### 2.4. Effect on Hemodynamic Parameters

**Figure 1:**
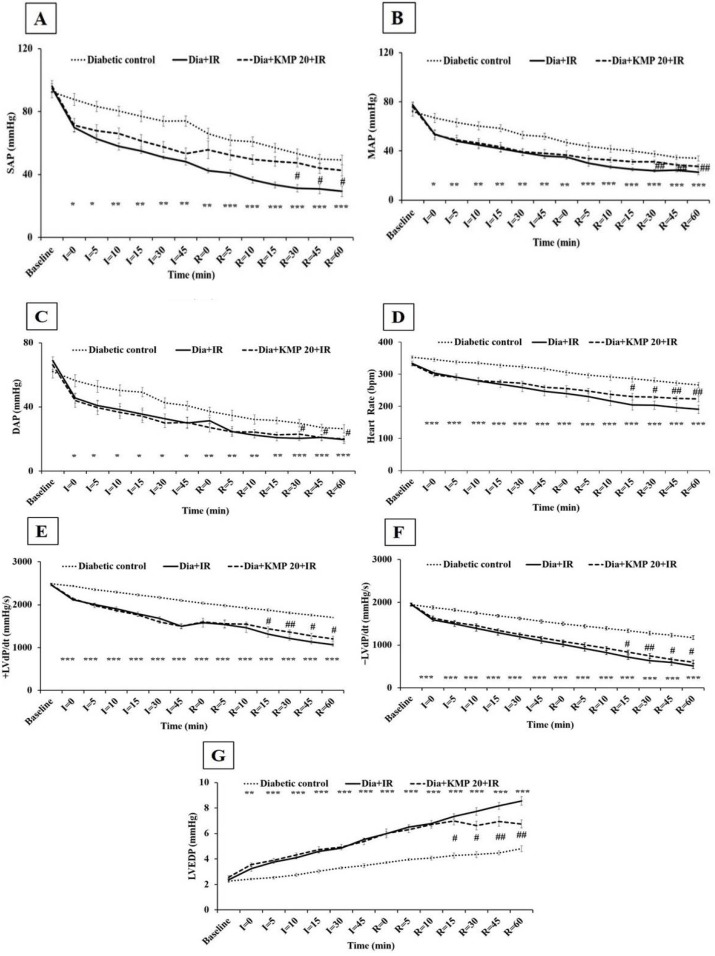
Effect of kaempferol on (**A**) systolic arterial pressure (SAP); (**B**) mean arterial pressure (MAP); (**C**) diastolic arterial pressure (DAP); (**D**) heart rate (HR); (**E**) maximal positive rate of the left ventricular pressure (+LVdP/dtmax); (**F**) maximal negative rate of the left ventricular pressure (−LVdP/dtmax) and (**G**) left ventricular end diastolic pressure (LVEDP). Data are expressed as the mean ± S.E.M.; *n =* 6 in each group. * *p* < 0.05, ** *p* < 0.01, *** *p* < 0.001 vs. Diabetic control; ^#^ *p* < 0.05, ^##^ *p* < 0.01 vs. Dia + IR.

Figure 1 demonstrates the effect of treatment on arterial pressure [systolic arterial pressure (SAP), mean arterial pressure (MAP), diastolic arterial pressure (DAP)], heart rate (HR) and left ventricle (LV) functions [±LVdP/dt and left ventricular end diastolic pressure (LVEDP)] during IR injury in diabetic rats. The diabetic IR group showed significant (*p* < 0.001) reduction of arterial pressure (SAP, MAP, DAP) and HR. In addition, there was significant ventricular dysfunction exhibited through decreased ventricular contraction (+LVdP/dt) and relaxation (−LVdP/dt) and increased preload (LVEDP) as compared to the diabetic-control group (*p* < 0.001). In comparison to the diabetic control group, kaempferol pre-treatment (20 mg/kg; i.p.) showed no significant improvement on cardiac function during the ischemia-reperfusion period. However, there was significant improvement in arterial pressure, heart rate, and ventricular functions in the diabetic rats at the end of the reperfusion period.

### 2.5. Effect on Biochemical Parameters

IR injury in diabetic rats resulted in significant decreases in GSH (*p* < 0.001), SOD (*p* < 0.01), and CAT (*p* < 0.01) antioxidant levels with concomitant increases in the level of MDA (*p* < 0.001), a by-product of lipid-peroxidation, and cardiac injury markers such as creatine kinase-MB isoenzyme (CK-MB) and lactate dehydrogenase (LDH) (*p* < 0.001) as compared to the diabetic control rats. Interestingly, kaempferol treatment markedly preserved the antioxidant status and thus normalization of the above mentioned biochemical parameters were recorded (Table 3).

### 2.6. Effect on Myocardial Apoptosis

**Figure 2:**
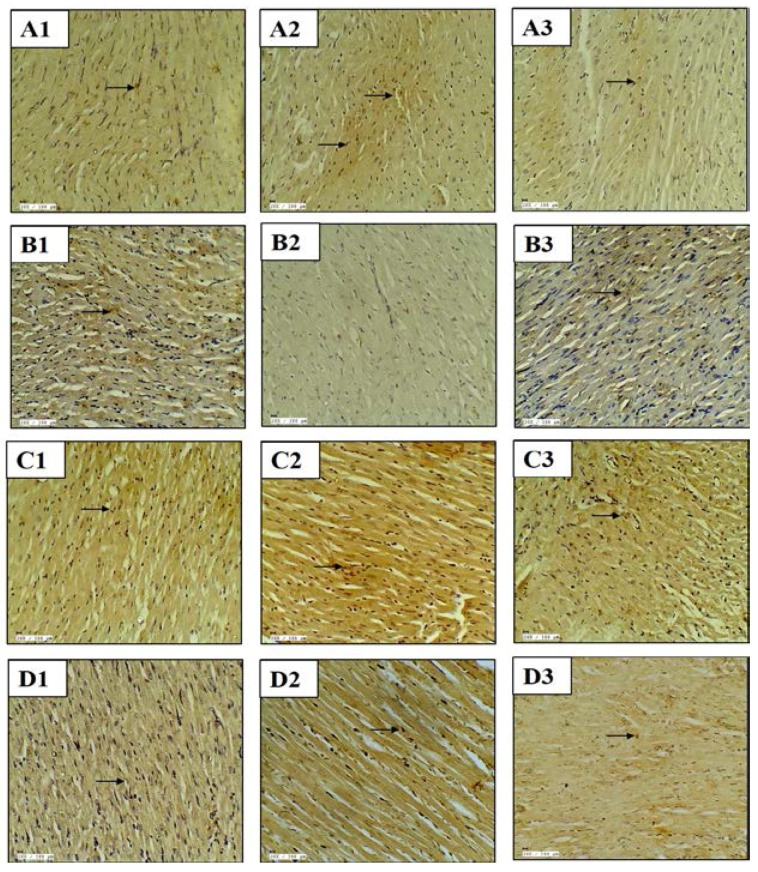
Effect of kaempferol on (**A1**–**A3**) Bax immunohistochemistry; (**B1**–**B3**) Bcl-2 immunohistochemistry; (**C1**–**C3**) Caspase-3 immunohistochemistry; (**D1**–**D3**) TUNEL assay. (**A1**–**D1**) diabetic control; (**A2**–**D2**) diabetes + ischemia-reperfusion and (**A3**–**D3**) diabetes + kaempferol 20 mg/kg + ischemia-reperfusion; *n =* 3 in each group; 20×; scale bar 100 μm; (→): positive stain.

The role of apoptosis in myocardial injury was assessed by studying the expression of anti and pro-apoptotic proteins by immunohistochemistry. There was increased expression of pro-apoptotic proteins (Bax and Caspase-3) and decreased expression of an anti-apoptotic protein (Bcl-2) in the myocardium of the diabetic-IR group. Concurrently, the TUNEL assay was also performed to detect DNA fragmentation in the nuclei. In the diabetes + IR group, there was an increased number of TUNEL positive nuclei. On the contrary, kaempferol pretreatment decreased the expression of pro-apoptotic protein and TUNEL positive cells and increased the expression of anti-apoptotic protein, and thus attenuated the apoptotic change observed in the myocardium (Figure 2).

### 2.7. Effect on Inflammatory Markers

The diabetes + IR group had significantly (*p* < 0.001) high levels of TNF-α and IL-6 as compared to the diabetic-control group. As anticipated, kaempferol treatment significantly reduced TNF-α (*p* < 0.01) and IL-6 (*p* < 0.05) levels in the serum (Table 4).

**Figure 3:**
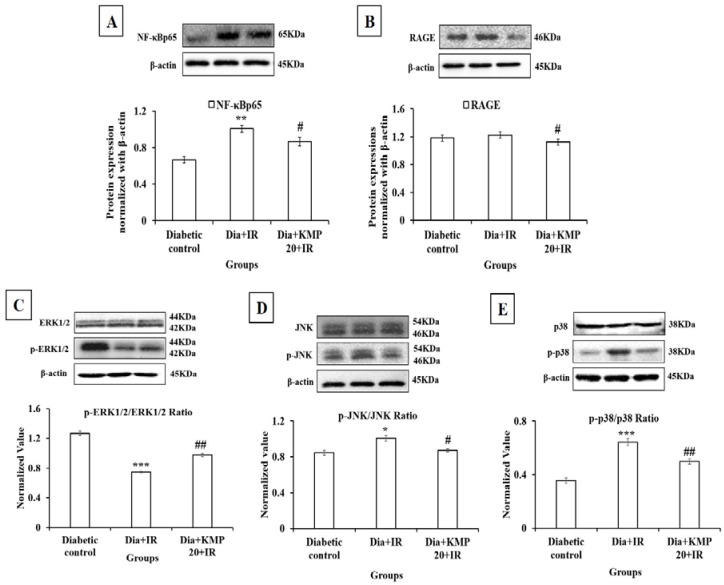
Effect of kaempferol on (**A**) NF-κBp65; (**B**) RAGE; (**C**) ERK1/2, p-ERK1/2; (**D**) JNK, p-JNK; (**E**) p38, p-p38. Protein expressions are normalized with β-actin. All the values are expressed as mean ± S.E.M.; *n =* 3 in each group. * *p* < 0.05; ** *p* < 0.01, *** *p* < 0.001 vs. diabetic control; **^#^** *p* < 0.05; **^##^** *p* < 0.01 vs. Dia + IR.

The activation of nuclear factor-kappa B (NF-κB) causes the release of inflammatory cytokines. Thus, Western blot analysis of NF-κBp65 was performed in the myocardium. In the diabetes + IR group, there was a significant (*p* < 0.01) increase in the expression of NF-κBp65 in the myocardium in comparison to the diabetic control group. Intriguingly, in the kaempferol pretreatment group we observed significantly decreased NF-κBp65 expression (*p* < 0.05). This suggests that kaempferol markedly suppressed inflammatory signaling (Figure 3).

### 2.8. Effect on Advanced Glycation End Products-Receptor for Advanced Glycation End Products (AGE-RAGE) Axis

There was increased serum levels of AGEs in the diabetic control and diabetic-IR groups. The increased AGE will bind to its receptor, i.e., RAGE, thus there was increased levels of RAGE in the diabetic control and diabetes + IR groups. Treatment with kaempferol for 28 days significantly suppressed the AGEs (*p* < 0.05)-RAGE (*p* < 0.05) activation (Table 4 and Figure 3).

### 2.9. Effect on Mitogen Activated Protein Kinase (MAPK) Pathway

In the diabetic-IR group, there was significantly increased phosphorylation of JNK (*p* < 0.05) and p38 (*p* < 0.001) (stress activated pathway) and decreased phosphorylation of ERK1/2 (pro-survival kinase pathway) (*p* < 0.001) in the myocardium as compared to the diabetic-control group. Increased phosphorylation of JNK and p38 further caused the activation of apoptotic and inflammatory pathways. On the contrary, kaempferol (20 mg/kg) pretreatment significantly normalized the phosphorylation of the MAPK pathway, i.e., there were significantly decreased levels of p-JNK and p-p38, and higher levels of p-ERK1/2 in comparison to the diabetes + IR group. Thus, in the treatment group, kaempferol prevented inflammation and apoptosis in the myocardium of diabetic rats (Figure 3).

### 2.10. Effect on Histopathological and Ultrastructural Examination

**Figure 4:**
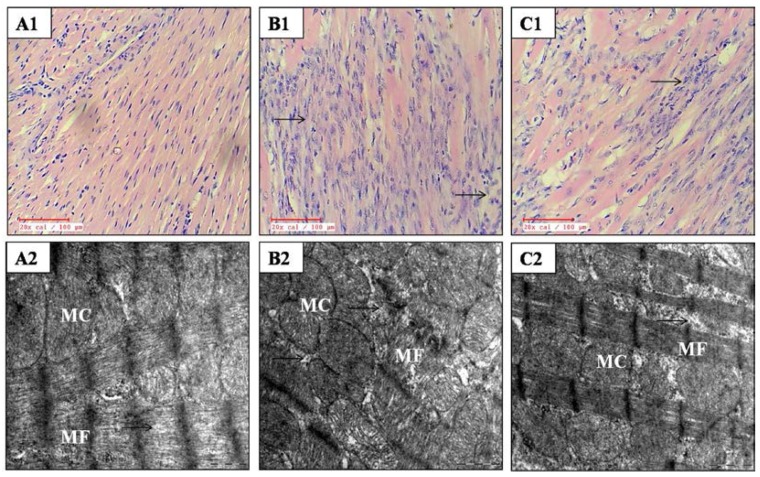
Effect of kaempferol on light microscopy (20×; *n =* 3 in each group) and electron microscopy (scale bar: 1 µm; *n =* 3 in each group). (**A1**,**A2**) diabetic control; (**B1**,**B2**) diabetes + ischemia-reperfusion and (**C1**,**C2**) diabetes + kaempferol 20 mg/kg + ischemia-reperfusion; MF: myofibrils; MC: mitochondria; (→): myocyte damage.

Figure 4 and Table 5 demonstrate the effect of kaempferol on histological alterations in IR insulted myocardium in diabetic rats. In the diabetes + IR group, the myocardium displayed marked membrane damage, myonecrosis, edema, infiltration of inflammatory cells, and a higher histological score. Kaempferol treatment preserved the myocardial architecture as there was reduced inflammation and edema. Also, the kaempferol treatment group also exhibited a low histological injury score as compared to the diabetes + IR group.

On ultrastructural examination, diabetes + IR rats revealed the disruption of cristae, and swollen and irregular mitochondria with nuclear and chromatin condensation in the myocardium. However, kaempferol treatment maintained the structural integrity. There was only mild mitochondrial swelling without any nuclear and chromatin condensation in rats treated with kaempferol (Figure 4).

## 3. Discussion

It is well known that diabetes is an important risk factor that predisposes to MI. It is also considered as one of the seven major controllable risk factors for heart disease. Diabetic adults are 2–4 times are more likely to develop cardiovascular diseases as compared to non-diabetic adults [20]. Cardiovascular disease death rates due to stroke and MI in the United States are 1.7 times more in diabetic adults than non-diabetic adults [21]. Furthermore, the relative risk for the occurrence of CVD ranges from 1–3 in men and from 2–5 in women with diabetes than those without diabetes [22]. Hence, the present study was undertaken to evaluate the potential of kaempferol in mitigating cardiac injury in diabetic rats subjected to IR injury. This study, thus, evaluates whether kaempferol can reduce cardiovascular mortality in the case of pre-existing diabetes. The results of our study support our hypothesis that kaempferol may indeed exert significant cardioprotection in this scenario. The inhibition of the AGE-RAGE/MAPK pathway by kaempferol along with its antioxidant, anti-inflammatory, and anti-apoptotic activity plays a significant role in its observed cardioprotective effect.

Kaempferol has been shown to exert beneficial effects through the modulation of the MAPK pathway [23]. MAPK plays a central role in the progression of cardiovascular diseases. In our study, kaempferol mediated modulation of the MAPK pathway was important in attenuating myocardial IR injury in diabetic rats. Furthermore, it also suppressed the AGE-RAGE axis. Thus, both these actions together resulted in improved cardiac function and reductions in oxidative stress, inflammation, and apoptosis in the diabetic rats.

Various studies have shown that AGE-RAGE axis is involved in the development of diabetic complications such as diabetic nephropathy, retinopathy, neuropathy, and cardiomyopathy [24,25,26,27]. Furthermore, in vivo studies have also shown the role of AGE-RAGE in myocardial ischemia reperfusion injury in diabetic rats [8]. In our study, kaempferol treatment also reduced hyperglycemia and prevented AGE-RAGE activation. Previously, various studies have demonstrated the anti-diabetic effect of kaempferol. An in vitro study has reported that kaempferol improved chronic hyperglycemia induced pancreatic β-cells [28]. Another study by Al-Numair et al. (2015) reported that kaempferol reduced the blood glucose level and attenuated oxidative stress in streptozotocin (STZ)-induced diabetic rats [16]. A previous study demonstrated that dietary intake of kaempferol significantly reduced hyperglycemia, hyperinsulinemia, and the circulating lipid profile level, and thus improved peripheral insulin sensitivity in obese diabetic mice [19]. Recently, one study has shown that kaempferol attenuated insulin resistance via reducing hepatic NF-κB/IKKβ signaling in type 2 diabetic rats [29]. Thus, blood glucose control may be a putative mechanism through which the inhibition of AGE-RAGE axis activation was achieved.

Previous studies have also demonstrated that AGE-RAGE promotes excess synthesis of highly reactive oxygen species (ROS) and causes oxidative stress that further exacerbates the progression of diabetes and its complications [30]. A study by Coughlan et al. (2009) linked AGE-RAGE to mitochondrial superoxide production with an increased mitochondrial permeability transition and showed that these effects were pronounced in diabetic rodents [31]. Mitochondrial oxidative stress, a consequence of IR injury, leads to the release of cytochrome C and activation of the cell death apoptotic pathways [32]. Our experiments revealed that there is an increased generation of oxygen rich free radicals, which disrupts the balance between oxidants-antioxidants. In our study, an IR challenge to the diabetic rats resulted in depletion of endogenous antioxidants GSH, SOD, and CAT. Furthermore, the presence of oxidative stress in the myocardium was evidenced by the increased MDA levels. MDA is a marker of lipid peroxidation and an increase in its levels is suggestive of the loss of membrane integrity and release of various cellular components like cardiac enzymes such as LDH and CK-MB from the intracellular compartment to the extracellular fluid. Pre-treatment with kaempferol for 28 days suppressed RAGE expression, boosted the endogenous antioxidant defense system, preserved membrane integrity, and prevented the release of CK-MB and LDH into the extracellular fluid. Thus, the mechanism by which kaempferol improved cardiac function may be related to RAGE mediated oxidative stress suppression. This is in accordance with other studies which have established its antioxidant effect in various disease models [16,33,34].

A large number of studies support the concept of RAGE mediated MAPK activation [35,36]. ERK1/2 (a pro-survival kinase), JNK, and p38 (stress activated protein kinases) are serine/threonine kinases that are part of the MAPK pathway [37]. ERK1/2 is involved in cell proliferation, differentiation, and cell survival [38]. Various studies have documented that activation or phosphorylation of ERK1/2 attenuates myocardial ischemia reperfusion injury and pharmacological inhibition of ERK1/2 abolishes the cardioprotection [39,40]. Ischemia and hypoxia are among the multiple factors that activate p38 and JNK and their activation leads to apoptosis and inflammation [41,42]. Previous studies have demonstrated that p38 phosphorylation increased during ischemia-reperfusion injury and the inhibition of phosphorylated p38 MAPK attenuated cardiomyocyte apoptosis following IR injury [40,43]. In another study, Li et al. (2014) reported that total flavones of *Choerospondias axillaries* improved myocardial IR injury due to antioxidant and anti-apoptotic effects that were attributed to the inhibition of phosphorylation of p38 and JNK [44]. Furthermore, Shi et al. have reported that N-acetyl cysteine attenuated human corneal epithelial cell apoptosis by inhibiting AGE induced phosphorylation of p38 MAPK and JNK [45]. Similarly, in line with these studies, we also observed increased expression of p38 and JNK, caspase-3 and Bax, and decreased expression of ERK1/2 and Bcl-2 in the diabetic-IR myocardium. Xiao and colleagues have previously demonstrated the anti-apoptotic effect of kaempferol in vitro and in vivo in the setting of doxorubicin induced cardiotoxicity [18]. Treatment with kaempferol modulated the MAPK activation and reduced apoptosis in the diabetic rats. Thus, the modulation of the MAPK pathway by kaempferol is one of the mechanisms through which it exerts cardioprotection in the diabetic rats.

The association of inflammation with myocardial IR injury is well known [46]. In this study diabetic-IR rats demonstrated increased levels of inflammatory markers such as TNF-α, IL-6, and NF-κB in the myocardium. Interestingly, treatment with kaempferol inhibited the activation of NF-κB, a transcription factor, which further prevented the release of inflammatory cytokines. The possible explanation for this effect could be inhibition of the MAPK pathway through AGE-RAGE activation, which reduced the levels of inflammatory markers [46,47]. These molecular changes were further supported by inflammatory and necrotic changes in the histopathological examination of the tissue. There was marked edema, myonecrosis, and infiltration of inflammatory cells in IR diabetic rats. Pre-treatment with kaempferol decreased the expression of inflammatory markers and normal morphological structure preservation was observed in the pre-treated rats. In a recent study, Luo et al. (2015) demonstrated that kaempferol suppressed inflammatory lesions in diabetes by reducing TNF-α and IL-6 levels along with the reduced expression of IκB kinase (IKK) and the subsequent inhibition of the NF-κB pathway activation [29]. Thus, the findings from the current study propose that kaempferol can serve as a novel therapeutic strategy for the attenuation of inflammation associated with myocardial IR injury in the setting of diabetes.

In conclusion, the pre-treatment of diabetic rats with kaempferol significantly ameliorated IR-induced myocardial injury by normalization of hemodynamic parameters, maintaining oxidant-antioxidant status, reducing inflammation and apoptosis, and by inhibiting the MAPK/AGE-RAGE pathways. Thus, kaempferol has a beneficial effect in the treatment of myocardial injury with co-existing diabetes. Nevertheless, further clinical studies on kaempferol are required to prove its effectiveness in myocardial injury with diabetes.

## 4. Materials and Methods

### 4.1. Experimental Animals

The experimental protocol was approved and reviewed by the institutional animal ethics committee (717/IAEC/13) and conformed to the Indian National Science Academy guidelines for the use and care of experimental animals in research. Adult male albino Wistar rats (10–12 weeks old; 150–200 g) were procured from the Central Animal House Facility of the All India Institute of Medical Sciences. Animals were kept in the departmental animal house under standard temperature (25 ± 2 °C), relative humidity (60 ± 5%), and with a 12 h light/dark cycle. They had free access to food (Ashirwad Industries Ltd., Chandigarh, India) and water *ad libitum*.

### 4.2. Drugs and Chemicals

Kaempferol and Streptozotocin (STZ) were obtained from Sigma Aldrich, St. Louis, MO, USA. For administration, kaempferol was dissolved in 0.5% dimethyl sulfoxide (DMSO). All the chemicals used were of analytical grade. A one touch Ultra 2 Blood Glucose Meter for measuring blood glucose level was purchased from LifeScan, Inc., Milpitas, CA, USA. Creatine Kinase-MB (CK-MB) isoenzyme and Lactate dehydrogenase (LDH) kits were obtained from Spinreact, Girona, Spain and Logotech Private Limited, Delhi, India, respectively. ELISA kits for rat tumor necrosis factor-α (TNF-α), interleukin-6 (IL-6), and AGEs were procured from Diaclone Tepnel Company, Manchester, UK and RayBiotech, Inc., Norcross, GA, USA and Kowain Biotech Pvt. Ltd., Shanghai, China, respectively. The terminal deoxynucleotidyl transferase dUTP nick end labeling (TUNEL) assay kit was purchased from Biovision Inc., Milpitas, CA, USA. Primary antibodies for ERK1/2, phospho (p)-ERK1/2 (p-ERK1/2), c-Jun N-terminal kinase (JNK), p-JNK, NF-κBp65, caspase-3, and β-actin were obtained from Cell Signaling Technology, Danvers, MA, USA. Antibodies for Bcl-2, Bax, and p38 were purchased from Abcam, Cambridge, UK. Phospho p38 (p-p38) and RAGE antibodies were procured from Santa Cruz, California, USA. Secondary antibodies were purchased from Merck Genei, Delhi, India. All other chemicals used were of analytical grade.

### 4.3. Experimental Protocol

#### 4.3.1. Induction of Diabetes

On day 1, diabetes in overnight fasted rats (*n* = 40) was induced by administering a single injection of STZ (70 mg/kg; i.p.). For administration, STZ was freshly prepared in 0.1 M citrate buffer (pH 4.5). Since STZ degrades quickly in aqueous solution, for the best experimental results, it was prepared only for two rats at a time [48]. After three days of STZ administration, blood was withdrawn from the tail vein and the fasting blood glucose level (FBG) was estimated using the blood glucose meter. In our study, the induction rate for the development of diabetes was 85% as 34 out of 40 rats successfully developed diabetes FBG >250 mg/dL. The reason for non-occurrence of diabetes in all the rats could be due to degradation of the STZ solution or incorrect i.p. administration of STZ to those rats. Furthermore, the rats that developed diabetes remained diabetic until the end of the study. The diabetic rats were used further in the study. The test drug, kaempferol (20 mg/kg; i.p.) was administered to diabetic rats for 28 days. The kaempferol treatment commenced on the 4th day post STZ administration.

We had already screened three doses of kaempferol i.e., 5, 10, and 20 mg/kg; i.p. for their cardioprotective activity in the isoproterenol-induced model of myocardial necrosis in rats on the basis of hemodynamic, biochemical, and morphological parameters. Kaempferol at the dose of 20 mg/kg; i.p. exerted a significant cardioprotective effect as compared to the two lower doses, 5 and 10 mg/kg [49]. Furthermore, kaempferol (20 mg/kg) was also effective in the ischemia-reperfusion induced model of myocardial injury in rats [50]. Thus, kaempferol 20 mg/kg was selected for further evaluation.

#### 4.3.2. Ischemia-Reperfusion (IR) Induced Model of MI in Diabetic Rats

A total of 34 diabetic rats were distributed into 3 groups:

Group 1 (Diabetic control; *n* = 10): 0.5% DMSO (2 mL/kg/day; i.p.) was administered to rats for a period of 28 days. On the 28th day, a thread was passed beneath the left anterior descending (LAD) coronary artery but was not occluded.
Group 2 (Diabetes + IR; *n* = 12): 0.5% DMSO (2 mL/kg/day; i.p.) was administered to diabetic rats for a period of 28 days. On the 28th day, the LAD coronary artery was ligated for 45 min followed by reperfusion for 60 min.
Group 3 (Diabetes + Kaempferol 20 + IR; *n* = 12): Kaempferol (20 mg/kg/day; i.p.) was administered to diabetic rats for a period of 28 days. On the 28th day, the LAD coronary artery was ligated for 45 min followed by reperfusion for 60 min.

### 4.4. Assessment of Cardiac Function

On the 28th day, rats were anesthetized with pentobarbitone sodium (60 mg/kg; i.p.). The anaesthetized rat was then kept on an operating tray and its forelimbs were tied to the tray edges. This helped to keep the dorso-ventral part of the rat extended for surgery. The neck was opened with a ventral midline incision and a tracheostomy was performed. The rats were ventilated with room air from a positive pressure respirator (TSE animal respirator, Germany). The Y shape jugular vein was exposed and cannulated with a polyethylene tube attached to a three way cannula (Meditop Corporation (M) Sdn. Bhd, Selangor, Malaysia) and 0.9% normal saline was infused through it. The right carotid artery was also exposed and cannulated with a soft narrow heparinized bore arterial cannula and connected with a pressure transducer (Gulton Statham P231D, CA, USA) for monitoring hemodynamic parameters, i.e., systolic arterial pressure (SAP), mean arterial pressure (MAP), diastolic arterial pressure (DAP), and heart rate (HR).

After recording the hemodynamic parameters, a left thoracotomy was performed at the fifth intercostal space with the help of electric cautery to minimize blood loss during surgery. A normal saline soaked warm cotton gauge pack was used to keep the lungs wet and to keep them away from the operative field. The pericardium was incised longitudinally and the heart was exposed. A wide bore (1.5 mm) sterile metal cannula was inserted into the cavity of the left ventricle from the posterior apical region of the heart. The cannula was connected to a pressure transducer (Gould Statham P23ID) for recording the left ventricular pressure rate of contraction (+LVdP/dt); the rate of relaxation (–LVdP/dt) and pre load, i.e., the left ventricular end diastolic pressure (LVEDP) using the Biopac system software (BSL 4.0 MP36) (Biopac Systems Inc, Goleta, CA, USA). After the completion of the surgical procedure, blood was drawn from the heart and the rats were sacrificed with an overdose of pentobarbitone sodium (150 mg/kg; i.p.). The hearts were then excised and stored for biochemical, histopathological, and ultrastructural evaluation, immunohistochemistry (IHC), TUNEL assay, and Western blot analysis. Blood was centrifuged at 4000 rpm and was further used to assess IL-6, TNF-α, and AGEs levels and to estimate LDH and CK-MB isoenzyme activities.

### 4.5. Biochemical Studies

For biochemical estimation, the heart tissue was removed from liquid nitrogen, thawed at 4 °C, and weighed. A 10% tissue homogenate was prepared in ice-chilled phosphate buffer (0.1 M; pH 7.4) and part of this was used for the measurement of thiobarbituric reactive substances (TBARS) and the reduced glutathione (GSH) level. The rest of the homogenate was used for the estimation of superoxide dismutase (SOD), catalase (CAT) enzyme activities, and protein content.

The TBARS level was assessed in tissue homogenate as described by Ohkawa et al. (1979) [51]. To the tissue homogenate, 8.1% sodium dodecyl sulphate, 20% acetic acid (pH 3.5), and 1.5 mL of 0.8% thiobarbituric acid was added followed by heating at 95 °C for 60 min at 95 °C and cooling at room temperature. After cooling, a mixture of *n*-butanol:pyridine (15:1 *v*/*v*) was added, mixed thoroughly, and centrifuged at 5000 rpm for 15 min. The intensity of the pink color formed was read at 532 nm and expressed as nmole/g tissue wt.

For the GSH measurement, firstly tissue homogenate was mixed with an equal volume of 10% TCA. Then, it was centrifuged at 5000 rpm for 20 min and the supernatant thus obtained was used for the estimation of the GSH content. In the supernatant, Na_2_HPO_4_ (0.3 M; pH 8.0) and DTNB [5,5’-dithiobis-(2-nitrobenzoic acid)] were added in sequence. The absorbance was recorded at 412 nm. The GSH content is mentioned as µmole/g tissue wt. [52].

The SOD activity was determined according to the method of Marklund and Marklund (1974) [53]. Briefly, to the supernatant, phosphate buffer (0.1 M; pH 8.4) and pyrogallol (7.5 mM) were added. The absorbance was read at 420 nm for 2 min at 15 s intervals. One unit of enzyme activity is defined as the amount of enzyme required to produce 50% inhibition of pyrogallol auto-oxidation under the assay conditions and expressed as U/mg protein.

The CAT activity was measured using the method of Aebi (1984) [54]. To the tissue supernatant, phosphate buffer (50 mM; pH 7.0) and hydrogen peroxide (30 mM) were added. The absorbance was measured for 30 s at an interval of 5 s at 240 nm and written as U/mg protein.

Protein content in the tissue supernatant was determined by Bradford method (1976) [55]. To the supernatant, the Bradford reagent was added, vortexed, and the absorbance was read at 595 nm. The protein concentration in the sample was determined from a standard curve produced using different concentrations of Bovine Serum Albumin (BSA).

The CK-MB isoenzyme activity in serum was estimated using the Spinreact kit, Girona, Spain. To the serum, the working reagent was added and incubated for 10 min at room temperature. The initial absorbance was noted at 340 nm and the final absorbance was noted after a 5 min interval. The difference in absorbance was calculated and multiplied by 1651 to give the final CK-MB activity. The CK-MB activity in serum is presented as U/L.

The LDH activity in the serum was measured using a kit obtained from Logotech, Delhi, India. To the serum, reagents 1 and 2 were added and incubated for 1 min. The initial absorbance was recorded at 340 nm and the final absorbance was read after 4 min. The difference between absorbances was calculated, multiplied by 8095 to obtain the final LDH activity, and expressed as U/L.

### 4.6. Measurement of Tumor Necrosis Factor-α (TNF-α), Interleukin-6 (IL-6), and Advanced Glycation end Products (AGEs) Levels

For the estimation of serum TNF-α, the samples and standard were added into the wells. Then, biotinylated antibody was added into each well and incubated for 3 h at room temperature. Following this, streptavidin-HRP solution was added into each well and incubated at room temperature for 30 min followed by washing. Then, the TMB substrate solution was added and incubated at room temperature in the dark for 10 min. The absorbance was read at 450 nm using an ELISA reader (Biotek Instruments, Winooski, VT, USA). The standard curve was plotted and the TNF-α concentration in the sample was determined from the standard curve.

For IL-6, the samples and standard were added into the ELISA plate and incubated for 2.5 h at room temperature. Subsequently, the plate was washed, and the biotinylated antibody for IL-6 was added and incubated for 1 h at room temperature. Then, Streptavidin-HRP solution was added and incubated for 45 min at room temperature. Following this, chromogen was added into the wells and incubated for 30 min in the dark. The intensity of color development was read at 450 nm with an ELISA reader. The standard curve was plotted using different concentrations of the standard. The concentration of IL-6 present in the sample was calculated from the standard curve and expressed as pg/mL.

For assessment of the AGEs level in the serum, the samples and standard were added into the 96-well enzymatic plate. Then, streptavidin-HRP was added and incubated for 60 min at room temperature followed by washing. Chromogen solution was added and the plate was incubated for 10 min in the dark at room temperature. The absorbance was measured at 450 nm, the standard curve was plotted, and the concentration of the AGEs level was determined in the sample from the standard curve.

### 4.7. Histopathological and Ultrastructural Evaluation

After sacrificing, heart tissues were fixed in 10% neutral buffered formalin and then embedded in paraffin. Then, the tissue sections (5 µm thick) were cut thereafter using a microtome (Leica RM 2125, Nussloch, Germany) and stained with hematoxylin and eosin (H&E). At least three hearts from each group underwent histological examination under the light microscope (Dewinter Technologies, San Donato Milanese, Lombardia, Italy). These sections were graded for severity of damage using a score on a scale of (Absence) no change; (Mild) focal change; (Moderate) patchy change; (Severe) confluent change.

Ultrastructural evaluation was done by transmission electron microscopy (TEM). Tissues were fixed in Karnovsky’s fixative, washed in ice-chilled phosphate buffer (0.1 M, pH 7.4), and post fixed for 2 h in 1% osmium tetroxide in the phosphate buffer at 4 °C. Later, sections were embedded in araldite CY212 to make tissue blocks. Ultrathin tissue sections (70–80 nm) were cut with an ultramicrotome (Ultracut E, Reichert Jung, Vienna, Austria) and stained with uranyl acetate and lead acetate to visualize under a transmission electron microscope (Morgagni268D, FEICo., Eindhoven, The Netherlands).

### 4.8. Immunohistochemistry (IHC)

For IHC, tissue sections were deparaffinized and rehydrated by passing through xylene to a graded series of ethanol. For antigen retrieval, the sections were heated in a microwave at 95 °C for 10 min in citrate buffer (10 mM; pH 6.0). Then, to prevent endogenous peroxides and non-specific binding, the sections were incubated with 30% hydrogen peroxide (H_2_O_2_) in methanol for 30 min and with normal goat serum for 2 h, respectively. Following this, the sections were incubated with Bcl-2, Bax, and Caspase-3 for 48 h. These primary antibodies were detected with horse radish peroxidase (HRP)-conjugated secondary antibodies (Merck Genei) and incubated for 2 h. The colorimetric reaction was developed with the addition of DAB. Then sections were stained with hematoxylin and mounted with DPX to visualise under a light microscope (Dewinter Technologies, San Donato Milanese, Lombardia, Italy).

### 4.9. TUNEL Assay

Initially, the sections were passed through xylene and graded series of ethanol (to deparaffinize and rehydrate the tissue). Then, the sections were incubated with Proteinase K to enhance tissue permeability. Thereafter, 30% H_2_O_2_ was added to the sections (to reduce any endogenous peroxidase activity). Then, the sections were incubated with complete labeling reaction buffer and antibody solution. DAB was added to visualize the antigen-antibody reaction. Sections were then visualized and examined under a light microscope. From each group, at least 5 fields in each slide were observed for any TUNEL positivity. The investigator performing the histopathological, ultrastructural, and TUNEL evaluation was blinded to the treatment protocol.

### 4.10. Western Blot Analysis

For Western blot analysis, the heart tissue was removed from liquid nitrogen, thawed at 4 °C, and weighed. Tissue homogenate was prepared in RIPA (radio-immuno precipitation assay) lysis buffer containing 150 mM NaCl, 10% Triton X-100, 0.5% Sodium deoxycholate, 0.1% Sodium dodecyl sulphate, 50 mM Tris base, and a protease inhibitor cocktail (Sigma Chemicals Co., St. Louis, MO, USA). Then, the homogenate was centrifuged at 12,000 rpm for 20 min at 4 °C and the supernatant obtained was used for the estimation of protein content using the method described by Bradford (1976) [55]. A protein concentration equal to 40 µg was loaded into each well of the sodium dodecyl sulphate polyacrylamide gel electrophoresis (SDS-PAGE) and then transferred to the nitrocellulose membrane. The membrane was then incubated with primary antibodies, i.e., ERK1/2, p-ERK1/2, JNK, p-JNK, p38; p-p38, NF-κBp65, RAGE and β-actin overnight at 4 °C followed by incubation with HRP-conjugated secondary antibodies (Merck Genei) for 2 h at room temperature. Later, the primary-secondary antibody interaction was detected with an enhanced chemiluminescence (ECL) (Thermo Fisher Scientific Inc., Rockford, IL, USA) kit and quantified by using the ImageJ software.

### 4.11. Statistical Analysis

The data are presented as means ± S.E.M. The statistical analysis was performed with one way analysis of variance (ANOVA) followed by the Tukey-Kramer post-hoc test using the Graph Pad InStat 3.1 (Graph Pad Instat Software Inc., San Diego, CA, USA). *p* < 0.05 was considered as statistically significant.

## Figures and Tables

**Table 1:** Effect of kaempferol on body weight changes. Data are expressed as the mean ± standard error of mean (S.E.M).; *n =* 6 in each group. * *p* < 0.05 vs. baseline value in respective groups. Dia + IR: diabetes + ischemia-reperfusion; Dia + KMP 20 + IR: diabetes + kaempferol 20 mg/kg + ischemia-reperfusion.

Groups	1st Day	7th Day	14th Day	21st Day	28th Day
Diabetic control	153.58 ± 4.30	148.26 ± 5.86	144.27 ± 4.76	141.43 ± 2.18	134.04 ± 3.57 *
Dia + IR	150.55 ± 5.47	146.09 ± 4.34	141.70 ± 4.41	138.06 ± 3.66	132.32 ± 2.81 *
Dia + KMP 20 + IR	147.55 ± 3.92	142.41 ± 4.71	138.78 ± 3.73	134.85 ± 3.19	130.64 ± 2.88 *

**Table 2:** Effect of kaempferol on blood glucose level. Data are expressed as mean ± S.E.M.; *n =* 6 in each group. * *p* < 0.05 vs. Diabetic control and Dia + IR groups.

Groups	1st Day	7th Day	14th Day	21st Day	28th Day
Diabetic control	497 ± 13.23	495.17 ± 11.01	500.17 ± 10.74	501.83 ± 16.31	503.5 ± 10.99
Dia + IR	512.18 ± 15.40	507.05 ± 17.15	510.02 ± 18.56	503.13 ± 14.86	505.31 ± 11.88
Dia + KMP 20 + IR	493.95 ± 17.75	486.3 ± 13.60	478.45 ± 21.37	470.39 ± 14.64	459.20 ± 10.29 *

**Table 3:** Effect of kaempferol on oxidant-antioxidant parameters and cardiac injury markers. MDA: malondialdehyde; GSH: reduced glutathione; SOD: superoxide dismutase; CAT: catalase; LDH: lactate dehydrogenase; CK-MB: creatine kinase-MB isoenzyme. Data are expressed as mean ± S.E.M.; *n =* 6 in each group. ** *p* < 0.01, *** *p* < 0.001 vs. Diabetic control; ^#^ *p* < 0.05, ^##^ *p* < 0.01 vs. Dia + IR.

Groups	MDA (nmole/g Tissue)	GSH (μmole/g Tissue)	SOD (U/mg Protein)	CAT (U/mg Protein)	LDH (U/L)	CK-MB (U/L)
Diabetic control	67.19 ± 3.11	1.02 ± 0.07	4.35 ± 0.48	5.96 ± 0.56	505.94 ± 21.99	422.66 ± 23.72
Dia + IR	103.41 ± 4.05 ***	0.50 ± 0.05 ***	2.10 ± 0.51 **	3.22 ± 0.31 **	764.98 ± 28.40 ***	662.6 ± 25.59 ***
Dia + KMP 20 + IR	82.39 ± 2.46 ^##^	0.84 ± 0.06 ^##^	3.91 ± 0.35 ^#^	5.05 ± 0.37 ^#^	646.25 ± 25.46 ^##^	555.29 ± 14.50 ^##^

**Table 4:** Effect of kaempferol on serum tumor necrosis factor-α (TNF-α), interleukin-6 (IL-6), and advanced glycation end products (AGEs) levels. TNF-α: tumor necrosis factor-α; IL-6: interleukin-6; AGEs: advanced glycation end products. Data are expressed as mean ± S.E.M.; *n =* 6 in each group. *** *p* < 0.001 vs. Diabetic control; **^#^** *p* < 0.05, **^##^** *p* < 0.01 vs. Dia + IR.

Groups	TNF-α (pg/mL)	IL-6 (pg/mL)	AGEs (ng/mL)
Diabetic control	15.5 ± 0.83	19.06 ± 0.67	24.89 ± 0.66
Dia + IR	23 ± 0.97 ***	28.06 ± 0.77 ***	26.54 ± 1.29
Dia + KMP 20 + IR	17.58 ± 0.89 ^##^	23.62 ± 0.97 ^#^	22.40 ± 0.82 ^#^

**Table 5:** Effect of kaempferol on histopathological evaluation. The changes are ranked as: (Absence) no change; (Mild) focal change; (Moderate) patchy change; (Severe) confluent change; *n =* 3 in each group.

Groups	Necrosis	Edema	Inflammation
Diabetic control	Mild	Absence	Mild
Dia + IR	Severe	Moderate	Severe
Dia + KMP 20 + IR	Moderate	Absence	Mild
